# Protective role of autophagy in triptolide-induced apoptosis of TM3 Leydig cells

**DOI:** 10.2478/jtim-2021-0051

**Published:** 2022-05-05

**Authors:** Xiaoyun Ye, Liang Chen

**Affiliations:** Medical Center of Reproductive and Genetics, Peking University First Hospital, Beijing 100034, China

**Keywords:** autophagy, triptolide, TM3

## Abstract

**Background and Objectives:**

Triptolide (TP) is known to impair testicular development and spermatogenesis in mammals, but the mechanism of the side effects still needs to be investigated. The aim of the research is to confirm whether TP can cause autophagy in TM3 Leydig cells and the potential molecular pathway in vitro.

**Methods:**

TM3 Leydig cells are used to investigate the molecular pathway through Western blot, detection of apoptosis, transmission electron microscopy for autophagosomes and so on.

**Results:**

The data show that TP treatment resulted in the decreasing of the viability of TM3 cells due to the increased apoptosis. Treated with TP, the formation of autophagosomes, the decrease in P62, and the increase in the conversion of LC3-I to LC3-II suggested the induction of autophagy. The induction of autophagy has accompanied the activation of the mTOR/P70S6K signal pathway. The viability of the TM3 cells was further inhibited when they were co-treated with autophagy inhibitor, chloroquine (CQ).

**Conclusion:**

All these data suggest that autophagy plays a very important role in antagonizing TM3 cell apoptosis during the TP exposure.

## Introduction

Infertility is a growing problem and about 8%–12% of reproductive-aged couples suffer worldwide,^[1]^ with approximately 25%–30% having male-factor infertility.^[2]^ In a long-term clinical observation, it has been found that reproductive issues associated with herbal therapies have increased recently, especially in patients with autoimmune disorders and cancer.^[3]^

Triptolide (TP) is a major active ingredient of *Tripterygium wilfordii* Hook F,^[4]^ which exhibits various biological functions, including immunosuppression,^[5]^ antitumor activity,^[6]^ antiinflammatory,^[7]^ neurotrophic, and neuroprotective effects.^[8]^ However, TP has many side effects that can cause damage to multiple organs.^[9]^ Furthermore, its reproductive toxicity has been proven serious, which limits its clinical application.

It has been demonstrated that TP can reduce estradiol and progesterone levels and increase follicle-stimulating hormone (FSH) and luteinising hormone (LH) levels,^[10,11]^ even reduce the relative weights of the ovary and uterus, and increase follicular atresia.^[12]^ In male rats, TP is capable of reducing the weight of the testis and epididymis, the motility and viability of sperm,^[13]^ and increases instances of sperm deformity.^[14]^ TP has not been shown to cause pathological changes in Leydig cells or the epididymal epithelium.^[15]^ However, another study showed that TP exposure suppressed the marker enzymes of spermatogenesis and testosterone.^[14,16]^ In brief, TP not only affects female reproduction, but also causes spermatogenesis dysfunction.

Testosterone is a major androgen that plays an important role in maintaining normal sexual function, spermatogenesis, and fertility in adult males. Leydig cells, one of the somatic cells in the testis, are located in the seminiferous tubules. Ninety-five percent of androgens are secreted by Leydig cells in normal adult male individuals. The steroid-synthesizing acute regulatory (StAR) protein, located on the mitochondrial membrane of cells, plays an important role in regulating steroid hormone synthesis.^[17]^ The synthesis of steroid hormones requires precursor cholesterol to be transported from the mitochondrial outer membrane to the mitochondrial inner membrane. This is the limiting step in the synthesis of steroid hormones. After entering the mitochondria, cholesterol is cleaved by cytochrome P450 cholesterol side chain lyase and then hydroxylated to pregnenolone to synthesize testosterone.^[18]^ It has been reported that testosterone levels affect spermatogenesis.^[19]^ Abnormal spermatogenesis, a common pathophysiological process, is associated with male infertility. TP causes Leydig cell apoptosis,^[20]^ but whether it can cause testicular steroidogenic toxicity and further affect spermatogenesis requires further investigation.

Autophagy is a metabolic process that maintains cell homeostasis and prevents the accumulation of harmful mutations. It is accompanied by other forms of cell death, such as apoptosis.^[21]^ A previous review showed that TP can inhibit the proliferation of spermatogenic cells and induce cell apoptosis through oxidative stress, but whether TP acts on Leydig cells and its related mechanisms was not investigated. Based on these findings, we attempted to confirm whether TP can cause autophagy in TM3 Leydig cells and investigate the potential molecular pathway to explain how TP influences male infertility *in vitro*.

## Materials and methods

### Reagents

Cell culture medium, Dulbecco’s Modified Eagle Medium: F12 (DMEM/F12), and phosphate buffer solution (PBS) were purchased from Hyclone (USA). Fetal bovine serum was obtained from BI (Biological Industries, Israel), and TP was purchased from Beijing ChengZhiKewWei Biological Engineering (Beijing, China). Chloroquine (CQ) (cas: 5063-05), dimethyl sulfoxide (DMSO) (cas: 67-68-5), and monodansyl-cadaverine (MDC) were purchased from Sigma-Aldrich (USA). β-Actin (TA-09) and β-tubulin (TA-10) antibodies were purchased from Zhongshanjinqiao; anti-LC3B monoclonal antibody (#2775), anti-P62 monoclonal antibody (#8025), anti-mammalian rapamycin target protein (mTOR) monoclonal antibody (#2983), anti-p-mTOR monoclonal antibody (#5536) , anti-StAR monoclonal antibody (#8449), anti-p70s6k monoclonal antibody(#2708), anti-p-p70s6k monoclonal antibody (#9234) , anti-4E-BP1 monoclonal antibody (#9644) , and anti-p-4E-BP1 antibody (#2855) were purchased from Cell Signaling Technology (USA). The cell-counting kit (CCK)-8 and Annexin V-FITC/propidium iodide (PI) apoptosis assay kits were obtained from Kaiji Biological Technology (Nanjing, China).

### Cell culture

TM3 cells were obtained from Professor Haoshu Luo of China Agricultural University. DMEM/F12 enriched with 10% fetal bovine serum was used for the cell culture. All cells were maintained at 37°C in a humidified atmosphere containing 5% carbon dioxide.

### Cell viability assay

TP was dissolved in DMSO at a concentration of 200 mmol/L as a stock solution and stored at –20°C until use. To evaluate the toxic effect of TP on cell growth, TM3 cells were plated at a density of 4 × 10^3^ cells/well in 96-well culture plates. After the cell attachment density was approximately 50–60%, the medium was discarded and replaced with 100 μL of the fresh serum-free medium containing various concentrations (25, 50, 100, 150, and 200 nmol/L) of TP and five replicate wells were set in each group and cultured for 12, 24, 36, and 48 h. After the treatment, the cell viability was determined the CCK-8 assay. Briefly, the culture medium was removed and 100 μL of fresh serum-free medium containing 10% CCK-8 was added to each well and incubated for 1 h at 37°C in the dark. Absorbance was then measured at 450 nm.

### Detection of apoptosis

Apoptosis was quantified using an Annexin V-FITC/PI apoptosis kit. TM3 cells were seeded at a density of approximately 20%–30% on 6-cm plates. When the attachment density was approximately 60%–70%, the cells were treated with varying concentrations of TP (50, 100, and 200 nmol/L) or a control (0.1% DMSO). Twenty-four hours later, TM3 cells were rinsed once with PBS, digested with trypsin lacking EDTA, centrifuged at 800 rpm/min for 5 min, washed twice with cold PBS, and stained with 5 μL PI and 5 μL Annexin V-FITC in 500 μL of binding buffer in the dark at room temperature for 15 min. Cell apoptosis was determined using a FACScan flow cytometer (Becton-Dickinson, USA).

### Monodansyl cadaverine (MDC) staining

MDC can be used to specifically detect the formation of autophagic vacuoles in macrophages and acidic organelles. Chemical staining with MDC was used to detect the acidic vesicular organelles. TM3 cells were seeded at a density of 20%–25% in a 35-mm confocal glass bottom dish. After attachment density was approximately 60%–70%, the cells were treated with varying concentrations of TP (50, 100, and 200 nmol/L) or a control (0.1% DMSO) for 24 h. The medium was discarded and 2 mL complete medium containing 50 μmol/L monodansyl cadaverine was added and incubated for 30 min at 37°C in the dark, washed three times with PBS, and observed under a laser confocal microscope.

### Transmission electron microscopy (TEM) for autophagosomes as an evidence of autophagy

TEM is a common methods for monitoring autophagy. TM3 cells were plated at a density of approximately 20%–30% on 10-cm plates. After attachment density was approximately 60%–70%, the cells were treated with various concentrations of TP (50, 100, 200 nmol/L) or a control (0.1% DMSO) for 24 h. The cells were harvested, washed, and fixed with 1% glutaraldehyde at 4°C. Samples were further postfixed in 1% osmium tetroxide, dehydrated in serial acetone, and embedded in Epon 812 resin; the ultrathin sections were stained with uranyl acetate and lead citrate, and then observed and photographed under a transmission electron microscope (JEM-1230, JEOL, Tokyo, Japan) at 80 kV.

### Western blot analysis

TM3 cells were plated at a density of approximately 20%–30% on 6-cm plates. After attachment density was approximately 60%–70%, the cells were treated with various concentrations of TP (50, 100, and 200 nmol/L) of a control (0.1% DMSO) for 24 h. Cells were washed with precooled PBS, RIPA lysate was added, centrifuged at 12,000 rpm for 10 min at 4°C, and the supernatant was collected. The protein concentration was determined using the BCA method (Beyotime Biotechnology, China). The protein was adjusted to an equal concentration with bromophenol blue and a lysate, and denatured at a high temperature. Equal amounts of protein (20–40 μg/lane) were subjected to sodium dodecyl sulfate-polyacrylamide gel electrophoresis (SDS-PAGE). Subsequently, electrophoresis was performed at 80 V for 30 min and 120 V for 90 min, and then the gel was transferred to polyvinylidene difluoride (PVDF) membranes (Bio-Rad, USA). The membranes were blocked with 5% BSA or skim milk solution for 1 h at room temperature. The membranes were incubated overnight with monoclonal primary antibodies in Tris-buffered saline with 0.1% Tween20 detergent (TBST) solution. The following primary antibodies were used: β-actin, β-tubulin, LC3B, P62, p-mTOR, mTOR, p-p70s6k, p70s6k, p-4EBP1, 4EBP1 (1:1000 in TBST), and StAR (1:500 in TBST). After being washed three times with TBST, the membranes were incubated with secondary HRP-conjugated anti-rabbit/mouse IgG antibody (1:5000 in TBST) for 1 h at room temperature. Finally, the protein bands were visualized using an enhanced chemiluminescence detection system. The data were normalized to the corresponding internal reference β-actin or β-tubulin. Immunoblotting densitometry analysis was performed using ImageJ software version 1.4 . All samples were analyzed in triplicate.

### Autophagy inhibitor treatment

Chloroquine, a specific autophagy inhibitor, was prepared as a 50-mmol/L stock solution in DMSO. For analysis of autophagy inhibitors, cultured cells were cotreated with the inhibitor at a final concentration of 50 mmol/L with 0.1% DMSO for 24 h. Control cells were treated with 0.1% DMSO.

### Statistical analysis

Statistical analysis was performed using the statistical software SPSS version 24.0 (Chicago, IL, USA). All values are presented as the mean ± standard deviation. The data were analyzed using one-way ANOVA. Statistical significance was set at *P* < 0.05. All data were obtained from at least three independent experiments.

## Results

### TP inhibits activity of TM3 cells in vitro

**Figure 1:**
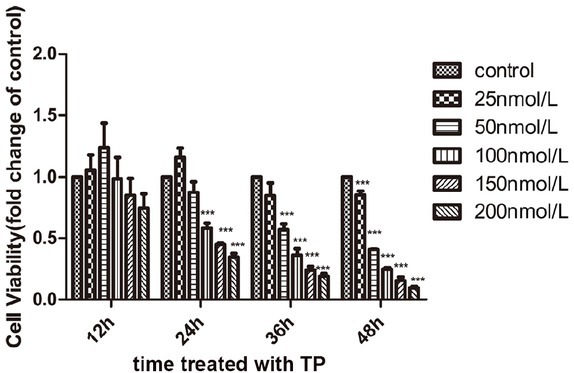
TP inhibits cell activity of TM3 cell. TM3 cells were treated with various concentrations of TP for 12, 24, 36, and 48 h or a control (0.1% DMSO) and then measured using CCK-8 proliferation assay. The results are expressed as mean ± SD (***P < 0.01 *vs*. control, *n* = 3). TP: triptolide; DMSO: dimethyl sulfoxide.

To determine the cytotoxicity of TP *in vitro*, TM3 cells were treated with TP at various concentrations for 12, 24, 36, and 48 h. Cell viability was measured using CCK-8 cell proliferation assay. As shown in Figure 1, TP inhibited TM3 cell growth in a dose- and time-dependent manner. When cells were exposed to 100 nmol/L TP for 24 h, the cell viability was 58%, resulting in a significant decrease compared to the control group. At the maximum concentration tested (200 nmol/L), the cell viability decreased to 74%, 35%, 19%, and 10% at 12, 24, 36, and 48 h, respectively, indicating that the cytotoxicity of TP is time-dependent. Results represent the average of three independent experiments.

### TP induces apoptosis of TM3 cells in vitro

**Figure 2:**
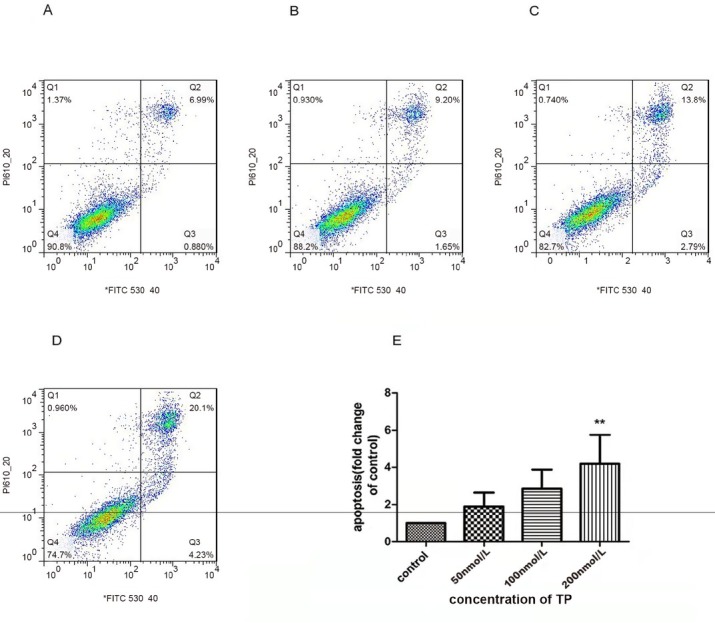
Effects of TP on cell apoptosis in the TM3 cells. A–D. Apoptosis in TM3 cells was detected after 24 h of treatment with TP at various concentrations (B: 50 nmol/L; C:100 nmol/L; D: 200 nmol/L) or a control (A : 0.1% DMSO) using Annexin V-FITC/PI binding and measured using flow cytometry analysis. E: The chart illustrates apoptosis proportion from three independent experiments (**P < 0.05 *vs.* control group, *n* = 3). TP: triptolide; DMSO: dimethyl sulfoxide.

To elucidate the mechanism by which TP causes cell death in TM3 cells, we monitored one of the apoptosis markers, Annexin, using V-FITC/PI staining. Cells were treated with various concentrations of TP for 24 h and then harvested for the apoptosis assay. The cytotoxic effects of TP were confirmed using flow cytometry. The cells in early apoptosis (Annexin V+/PI-) fell into the lower right quadrant, and the late apoptosis cells (Annexin V-/PI+) fell into the upper right quadrant. As shown in Figure 2A–2D, the apoptosis rate increased after treatment with TP (50, 100, and 200 nmol/L) for 24 h in a concentration-dependent manner. Our results also indicated that the apoptosis rate was significantly increased in response to 200 nmol/L TP compared to the control group (Figure 2E).

### Effect of TP on StAR expression of TM3 cell

**Figure 3:**
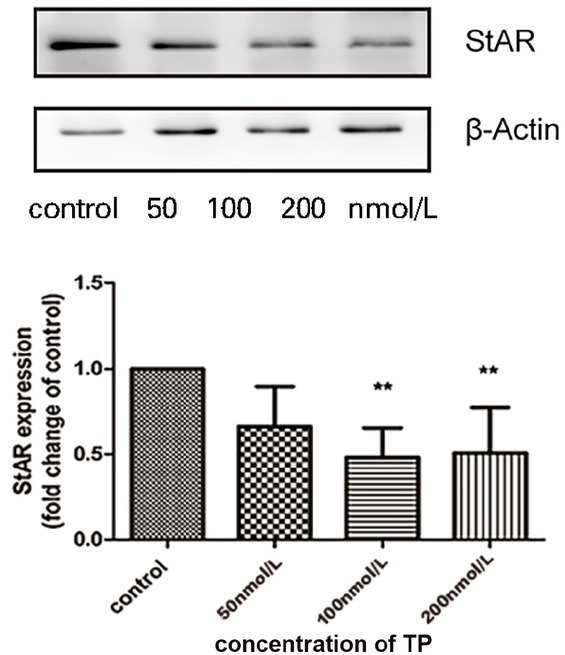
Effect of TP on StAR expression of TM3 cell. TM3 cells were treated with TP (50, 100, and 200 nmol/L) or a control (0.1% DMSO) for 24 h. Bar graphs are representative of three independent experiments (**P< 0.05 *vs.* control group, *n* = 3). TP: triptolide; StAR: steroid-synthesizing acute regulatory; DMSO: dimethyl sulfoxide.

TP inhibited StAR expression in TM3 cells. As shown in Figure 3, StAR expression in TM3 cells exposed to 100 or 200 nmol/L TP for 24 h significantly reduced compared to the control.

### TP increases autophagy of TM3 cell in vitro

The formation of autophagosomes during autophagy can be detected by classical methods, including MDC staining, western blotting, and electron microscopy.

**Figure 4:**
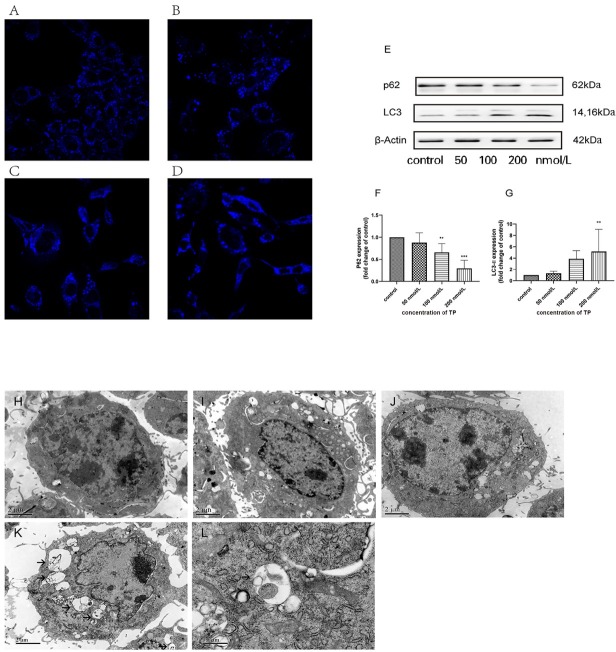
High concentration TP triggers autophagy in TM3 cells. A–D. To characterize autophagy, we used MDC staining to observe the acidic compartment in TP-treated TM3 cell. Cells were treated with various concentration for 24 h, (A) control, (B) 50 nmol/L, (C) 100 nmol/L, (D) 200 nmol/L. E. Expression of autophagy-related protein following treatment with TP (50, 100, and 200 nmol/L) for 24 h. F–G: Bar graphs are representative of three independent experiments. (**P < 0.05,***P < 0.01 *vs.* control group, *n* = 3). H–L. Ultrastructural evidence of TP-induced autophagy of TM3 cells. TM3 cells were treated with control (H), 50 nmol/L TP (I), 100 nmol/L TP (J), 200 nmol/L TP (K) for 24 h. Autophagic vacuoles were visualized using TEM (12,000× magnification). L. The classic structure (15,000× magnification) treated with 200 nmol/L TP. The autophagic vacuoles are indicated by arrows. S**cale bar: H-K=2** μ**m, L=0.5** μ**m. TP: triptolide; TEM: Transmission electron microscopy.**

MDC is a fluorescent dye that can be absorbed by cells and is displayed on autophagy vesicles. MDC-labeled bubble vesicles can be used to evaluate the level of autophagy in TM3 cells. MDC staining demonstrated that the treated groups exhibited higher fluorescence intensity and more MDC-labeled puncta compared to the control group. As shown in Figure 4A–4D, these results indicate that TP treatment increased autophagy in TM3 to some extent.

Furthermore, we evaluated the LC3-II and p62 protein levels by western blotting. The data (Figure 4E–4G)) showed that compared with the control group, the protein p62 decreased and the ratio of LC3-II/LC3-I increased with TP treatment (50, 100, and 200 nmol/L) for 24 h. These western blots were consistent with the fluorescence results, indicating that TP induced autophagy in TM3 cells.

Autophagy was morphologically defined by TEM in TM3 cells to confirm that it was induced by TP treatment. As depicted in Figure 4H–4L, TM3 cells treated with TP (100 and 200 nmol/L) exhibited several autophagosomes that were absent in the control cells. These results indicated that TP induced autophagy in TM3 cells.

### Effect of TP on the activation of mTOR signaling in cultured TM3 cells

**Figure 5:**
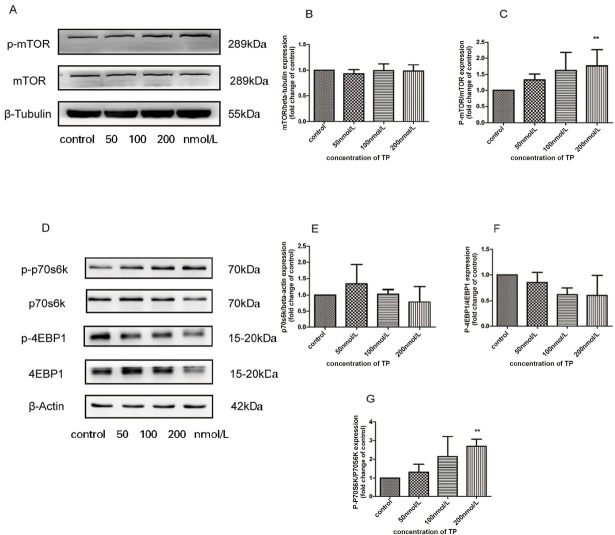
TP activates mTOR signaling pathway in cultured TM3 cells. Western blot analysis was used to detect the proteins expression.A: Expression of p-mTOR, mTOR protein following treatment with TP (50, 100, 200 nmol/L for 24 h.B-C: Bar graphs were representative of three independent expriments.D: Exrpession of p-p70s6k, p70s6k, p-4EBP1,and 4EBP1 protein following treatment with TP (50, 100, 200 nmol/L for 24 h.E-G: Bar graphs were representative of three independent expriments. Data were shown as mean±SD. (**P ﹤ 0.05 *vs.* control group, *n* = 3). TP: triptolide.

To assess the involvement of the mTOR signaling pathway in TP-induced apoptosis and autophagy of TM3, the expression levels of both total and phosphorylated proteins were evaluated by western blot analysis. TM3 cells were treated with various concentrations TP (50, 100, and 200 nmol/L) or a control (0.1% DMSO) for 24 h. As shown in Figure 5, we observed the activation of p-mTOR and p-p70s6k in a dose-dependent manner, and a significant difference was observed in 200 nmol/L group. However, we observed no change in the total mTOR and p70s6k concentrations after TP treatment. Suppression of p-4EBP1 was observed in TM3 cells, but there was no significant difference after treatment for 24 h.

### Autophagy inhibitor treatment

**Figure 6:**
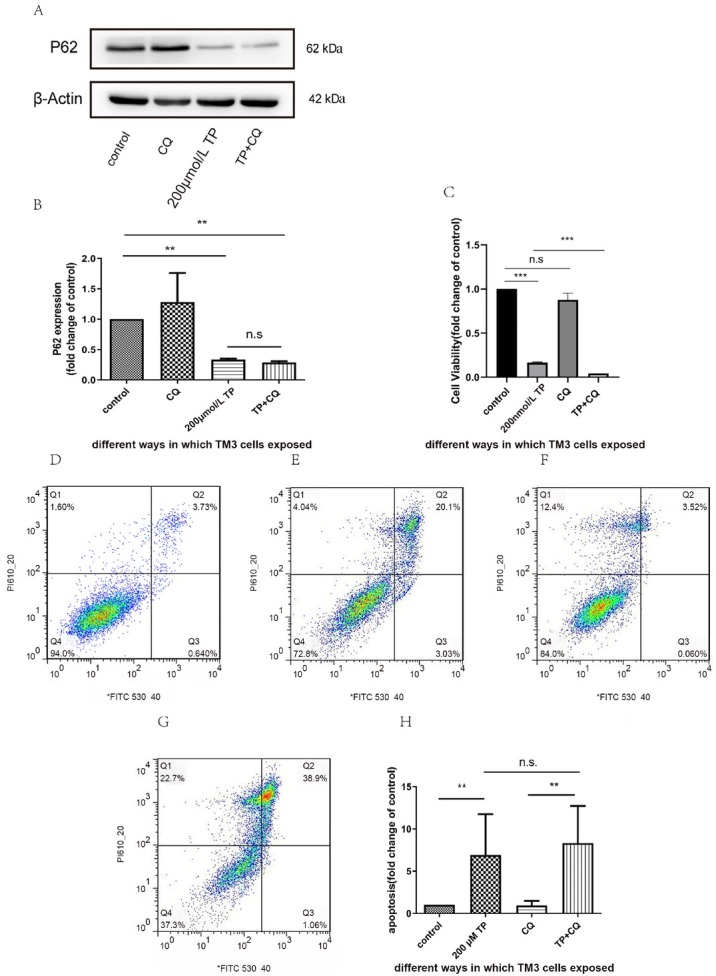
**Effects of CQ on TP-induced apoptosis. A–B: TM3 cells were pretreated with 50 nmol/L of CQ for 1 h, followed by 200 nmol/L TP treatment for 24 h. Western blot analysis and relative densitometry of P62 protein. Values are expressed as fold mean ± SD. C: Cell viability was measured using the CCK-8 assay. The results are presented as mean ± SD. D-H: TM3 cell were stained with Annexin V and propidium iodide, and the apoptotic was determined using flow cytometry (D: control, E: 200** μmol/L TP, F: CQ, G: TP+CQ,H:Bar graphs were representative of three independent expriments). The results were presented as mean±SD. **P ﹤ 0.05, ***P ﹤ 0.01 *vs.* control group, *n* = 3. CQ: chloroquine; TP: triptolide. n.s: no significant.

After exposure to the autophagy inhibitor CQ, we detected P62 protein by western blotting. As shown in Figure 6, no differences in p62 protein were apparent between the CQ and control groups. No significant difference was noted between the TP treatment and TP-CQ cotreatment groups. In addition, flow cytometry was used to detect apoptosis. No significant difference was observed in the CQ group compared to the control group. Although apoptosis increased in the TP-CQ cotreatment group compared to the TP treatment group, the difference was not statistically significant. CCK-8 was used to measure the cell viability, and no significant difference was observed between the CQ group and the control group. Compared with the TP group, cell viability was significantly reduced in the TP-CQ cotreatment group.

## Discussion

Autophagy is highly conserved during evolution and is a metabolic process in which eukaryotic cells degrade intracellular components through lysosomes, maintaining cell homeostasis and cell survival.^[22]^ Studies have confirmed that autophagy plays an important role not only in cancer, ^[23]^ neurodegeneration,^[24]^ and cardiovascular disease,^[25]^ but also in male reproductive dysfunction,^[26]^ especially in the formation of abnormal sperm.^[27]^ Spermatogenesis from the proliferation and differentiation of undifferentiated spermatogonial stem cells is based on the complete function of the tissue structure.^[28, 29, 30]^ In addition to supporting cells, Leydig cells play an important role in maintaining spermatogenesis^[31, 32, 33]^ and are affected by various chemicals and drug metabolism. ^[34, 35, 36, 37]^

TP is a widely used immunosuppressive agent that has adverse effects on the male reproductive system, as well as hepatotoxicity and nephrotoxicity.^[38]^ It has been reported that TP can reduce the cAMP content in HCG-induced cells and then disrupt the cAMP/protein kinase A (PKA)-mediated expression of estrogen synthase enzymes, leading to reduced estradiol synthesis.^[39]^ TP can bind to the active site of human estrogenic 17-β-hydroxysteroid dehydrogenase and human estrogen receptor.^[40]^ TP is observed on the inhibition of the mRNA expression of most genes related to steroigenesis.^[41,42]^ All that provide new views into the impact of steroidogenesis on the severe reproductive toxicity of TP. The steroidal toxicity of TP might be mainly due to disruption of direct cytotoxicity, which would lead to reduced sex steroid synthesis and reproductive dysfunction.^[42]^ However, there have been no reports of autophagy in the testes after TP exposure. We used TM3 cells as a model to explore the effects and molecular mechanisms of TP on male reproduction *in vitro*. In this study, we first investigated whether TP can induce autophagy activation in TM3 cells (Figure 4), which is accompanied by activation of the mTOR signaling pathway (Figure 5), and we found that inhibition of autophagy by CQ further reduced cell viability.

We found that TP not only inhibited the viability of TM3 cells, but also decreased the expression of StAR (Figure 3). These results are consistent with those of the previous studies reporting that inhibition of TM3 cell viability may affect testosterone expression.^[20]^ In addition, studies have shown that TP significantly inhibits cell viability at 50 nmol/L and significant apoptosis (Figure 2) at higher concentrations (200 nmol/L). Thus, there may be other mechanisms involved in regulating cell viability, and autophagy may be one of them. Studies have also shown that Leydig cell autophagy is involved in the synthesis and regulation of testosterone. ^[43]^

According to previous studies, microtubule-associated protein light chain 3 (LC3) is an important biomarker of autophagy and is present in two forms: the cytosolic, LC3-I, and the membrane-bound form, LC3-II.^[44]^ The cytosolic LC3-I is converted into LC3-II when the autophagy process is activated, so the level of autophagy can be measured by the amount of LC3-II. In this study, we found that TP could significantly increase the accumulation of LC3-II and accelerate the decomposition of P62, meanwhile the number of acidic vesicles increased. TEM was used as another method to identify autophagy (Figure 4), as this is ‘gold standard’ of autophagy identification. ^[44]^ Based on these results, we concluded that TP induced autophagy in TM3 Leydig cells.

To further explore the connections between cytotoxicity, autophagy, and apoptosis, we used CQ as an autophagy inhibitor which blocked the last step of the autophagy pathway.^[45]^ CQ, a lysosomal lumen alkalizer, blocks the autophagic process by impairing lysosomes.^[46]^ CQ has been reported to be the only clinically relevant autophagy inhibitor. It is widely used as an antimalarial and antirheumatic agent.^[47]^ CQ disrupts the pH value of acidic vesicles, prevents the fusion of autophagosomes with lysosomes, and subsequently impairs autophagic vesicle clearance. ^[47]^ TP-induced cell death was accelerated by CQ through inhibition of autophagy (Figure 6C), but apoptosis of TM3 Leydig cell toxicity could not be mitigated by autophagy (Figure 6D-H). This indicated that autophagy could protect cells, which is in agreement with previous research.^[48]^

Autophagy stabilizes the intracellular environment and maintains cell survival by synthesizing balanced anabolism and catabolism.^[49]^ The mTOR signaling pathway is also a major signal transduction cascade involved in cell metabolism, proliferation, and survival,^[50]^ and plays an important role in autophagy.^[51]^ Our results showed that the mTOR/P70S6K signaling pathway was activated (Figure 5). A previous study reported that the activation of mTOR phosphorylates the p70 ribosomal subunit protein S6 kinase p70S6K, which could promote protein synthesis.^[52]^ Studies have shown that activation of this pathway could have an antiapoptotic function through a series of phosphorylations.^[53]^ At the same time, some studies have confirmed that this pathway contributes to spermatogenesis^[55]^ and participates in the self-renewal of spermatogonial stem cells. ^[55]^ Therefore, we considered that activation of the mTOR signaling pathway is associated with abnormal cell survival conditions. In contrast, our results showed that activated autophagy had a protective effect on TP-induced cytotoxicity (Figure 6). To our knowledge, mTOR is an important negative regulator of autophagy, ^[56]^ and our results conflicted with this view. The signaling pathway is complex in regulating the development of autophagy. Perhaps TP-induced autophagy does not work through mTOR, but this requires further research.

## Conclusion

Our study demonstrates that TP inhibits cell viability and expression of StAR protein. It is the first time that TP has been reported to induce autophagy activation in TM3 cells and the autophagy plays a very important role in antagonizing TM3 cell apoptosis during TP exposure. This provides a novel molecular mechanism responsible for the regulation of TM3 cells following TP exposure.
